# Archives de Neurologie, Revue des Maladies Nerveuses et Mentales

**Published:** 1892-12

**Authors:** 


*Archives de Neurologie, Revue des Maladies Nerveuses et Mentales.* Publiée sous la direction de J.-M. CHARCOT. Paris: BUREAUX DU PROGRÉS MÉDICAL.

We are glad to welcome amongst our exchanges this very valuable publication, in which appear many of the most important contributions of French workers in neurological science. No one who desires to keep abreast of the advances made in neurology can afford to neglect this journal. In the present year we may mention, amongst many interesting contributions, papers "On the Association between Tabes Dorsalis and Diabetes," by MM. Guinon and Souques; "An Anatomical Study on the Arrangement of the Vessels in Nerves," by MM. Quénu and Lejars; "On a Case of General Paralysis commencing at a very early age," by MM. J.-M. Charcot and Dutil; and a very lucid and interesting study "On the Nature and Real Meaning of Hysterical Anæsthesia," by Prof. Janet. The rest of the journal is made up of good abstracts of papers from the current foreign journals, and by reviews and criticisms of works on neurological subjects.

